# Of Chorea with Aceto-Salicylic Acid

**Published:** 1907-02-02

**Authors:** 


### TREATMENT

### OF CHOREA WITH ACETO-SALICYLIC ACID.

It is always difficult to be sure whether any particular remedy is actually curative when administered in a given disease; but there seems to be little doubt about the good **results of** giving aceto-salicylic acid in cases of chorea. In a most interesting paper, read before the Therapeutical Society by Dr. Cecil Wall, the matter was fully dealt with recently. Dr. Wall's conclusions will probably be tested by large numbers of medical men in general practice.

Aceto-salicylic acid is, we are told, the chemical name for the active principle of a well-known proprietary medicine—aspirin. In order to test its efficacy in chorea, several hundreds of cases were dealt with. They were taken consecutively just as they came, mild or severe, without selection. Different methods of treatment were adopted for different consecutive batches of cases, and the basis for comparison of results was the duration of the movements. In all cases the general treatment, apart from drugs, was similar in the different batches, so that the results are comparable with one another.

When general measures alone were adopted, without drugs other than some iron preparation or possibly cod liver oil, the duration of the attack varied from 1 to 24 weeks, 30 per cent. lasting longer than 12 weeks and 52 per cent. longer than 8 weeks.

When arsenic was administered every known method of giving this remedy was tried, and the dosage was varied both upwards and downwards to the fullest extent; but the average duration of the attacks of chorea was little different to that of cases in which only iron or cod liver oil was given. Dr. Wall came to the conclusion that arsenic is very far from being the drug for chorea—at any rate in cases arising in or near London. It is possible that chorea in the North of England differs from chorea in the South, owing to differences of temperament in the patients or some other cause; for it was in the North that arsenic was first vaunted as the specific remedy for chorea.

When sodium salicylate was the drug given 42 per cent. of the cases remained choreic for more than 12 weeks, though 54 per cent. were cured in less than two months.

When aceto-salicylic acid (aspirin) was used no case continued choreic for more than 12 weeks, and in 92 per cent. of the cases the movements had ceased in less than two months. The relative values of the four lines of treatment are well brought out in this table:—

| | Cases treated with Arsenic. | Cases treated with Salicylate of Soda. | Cases treated with no particular Drug. | Cases treated with Aceto Salicylic Acid. |
|---|---|---|---|---|
| | % | % | % | % |
| Proportion of Cases cured in less than three months ... | 69 | 58 | 70 | 100 |
| Proportion of Cases cured in less than two months ...... | 38 | 54 | 48 | 92 |

The figures are sufficiently striking, and speak for themselves. They mean, if subsequent experience confirms them, that in aceto-salicylic acid we have a remedy which will enable choreic children to return to school about a month sooner all round than is possible when other remedies are employed.

In regard to the method of administration, the drug is given as a powder (not as pill or tabloid) in 10-grain doses three times a day for a child of 6; 10-grains four to six times a day for a child of 8 to 10; for a child of 12 or over, 20 grains every two hours for six doses, and then continued in 10 to 20-grain doses three times a day. Buzzing in the ears or vomiting are two of the least unlikely ill-effects, and hæmaturia has been observed in rare cases; but ill-effects of any kind are usually nil.

To some extent the aceto-salicylic acid acts in chorea as bromides do in epilepsy, or as sodium salicylate does in acute rheumatism; that is to say that whilst the symptoms subside comparatively rapidly under its use, they are apt to reappear if the drug be stopped too soon. It is best to continue with the aceto-salicylic acid for a fortnight or three weeks after the chorea has ceased; but the patient, provided there be no cardiac or other indication to the contrary, can return to school and lead an ordinary life meanwhile.

